# Determinants of Occupational Injury: A Case Control Study among Textile Factory Workers in Amhara Regional State, Ethiopia

**DOI:** 10.1155/2011/657275

**Published:** 2011-12-11

**Authors:** Zewdie Aderaw, Dagnew Engdaw, Takele Tadesse

**Affiliations:** ^1^Public Health Department, College of Health Sciences, Debre Markos University, P.O. Box 269, Debre Markos, Ethiopia; ^2^School of Public Health, University of Gondar, P.O. Box 269, Gondar, Ethiopia

## Abstract

*Background.* Occupational injuries pose major public health and socioeconomic developmental problems. However, efforts towards investigation of determinants among factory workers are very minimal in developing countries. Thus, this study aimed at to identify determinants of occupational injury among textile factory workers in Amahara regional state in Ethiopia. *Methods.* A case control study was done among 456 textile factory workers (152 cases and 304 controls). Self-reported data from workers and document review from factories clinics were used to ascertain occupational injury status within one-year period. Data was collected using pretested and structured questionnaire by trained data collectors. Odds ratio with 95% confidence interval was used to assess level significance. *Results.* Young age (<30 years) (AOR 1.90, 95% CI (1.22, 2.94)), male gender (AOR 2.54, 95% CI (1.58, 4.07)), health and safety training (AOR 1.85, 95% CI (1.17, 2.91)), sleeping disturbance (AOR 1.99, 95% CI (1.30, 3.04)), and job stress (AOR 2.25, 95% CI (1.15, 4.41)) were significant predictors of occupation injury. *Conclusion.* Lack of training, sleeping disturbance, and job stress increased the risk of occupational injury. So, providing basic health and safety training with special emphasis on younger and male workers, reducing stressors, and providing sleep health education were recommended.

## 1. Introduction

An occupational injury is any physical injury condition sustained on a worker in connection with the performance of his or her work in the industry. Employed people in industries spend at least one third of a day at work which have a strong effect on their health and safety due to work and work-related injuries [1]. These occupational injuries pose a major public health and developmental problems which result in a serious health, social, and economic consequences on workers and their employers [2, 3].

Worldwide in 2005, an estimated of 250 million occupational injuries and 5.4 million deaths due to injuries occurred annually. From this, over 90 percent was in low- and middle-income countries where the greatest concentration of world's workforce and low level of factories found [4]. This problem costs the world a loss of roughly 4% of the gross national product [5, 6]. Despite this, only 5 to 10 percent of the workforce in developing countries has access to some kind of occupational health and safety services [6].

 Ethiopia has been a member state of International Labor Organization and signed conventions related to health and safety of factory workers since 1923. However, the national occupational safety and health policy is not issued though it is required by the country as a result of ratifying occupational safety and health convention no. 155/1981 [7]. Currently to prevent occupational injury and to promote health and safety at work places, the Federal Ministry of Labor and Social Affairs, Amhara regional Bureau of Labor and Social Affairs and Affiliated Zonal representative offices have taken responsibilities for occupational safety and health services of workers according to labor proclamation no 377/2003 [8].

Studies done in France, USA and China indicated that men had a higher risk of occupational injury than women [2, 9, 10]. However, a study conducted in Ethiopia among small- and medium-scale factory workers indicated that occupational injury has no significant statistical association with gender of the worker [11]. Investigators at different places indicated that younger workers suffer more occupational injury at a higher rate than older workers [9, 12]. Also a study done showed that the prevalence of work and work-related injury increased with young age [11]. Most occupational health and safety studies conducted in developing countries revealed that increased educational levels in the factory have been associated with decreased work-related injuries [9–11, 13, 14].

Poor perception regarding to working conditions and safety environment had a significant influence on injury occurrence. Most researchers emphasize that work place injuries are caused by poor person environment which leads to increased job stress and, therefore, increases occupational injury risk [2]. A study done in Ethiopia North Gondar Zone among small- and medium-scale factory workers indicated that hours worked per week, workplace supervision, health and safety training showed a significant association with work and work-related injuries [11].

A study in India indicated that work accidents have been associated with alcohol consumption [15]. A study done in Ethiopia revealed that there was no significant association between khat chewing and cigarette smoking with occurrence of occupational injuries [11]. Most studies in different countries revealed that sleeping disorder, job stress and job dissatisfaction are the major risk factors for the occurrence of occupational injuries among industrial workers [14, 16]. A case control study among coal mining factory workers in India reported that workers who were highly satisfied with the existing jobs have lower risk of occupational injury. This can be explained that workers who did not injured have positive thinking about the physical environment and always take necessary safety precautions. This study also indicated that work injuries were caused by a poor person environment fit which leads to increased job stress. Such stresses increased occupational injury risks, and stressed individuals were more likely to have involved in occupational injuries [13]. A case control study done among Railway workers in France indicated that workers with sleeping disorder problem sustained more occupational injury compared with their counterparts [14].

In Ethiopia, different studies indicated that occupational injuries at manufacturing industries were highly significant [7, 11, 12, 17]. Amahara regional state Bureau of Labor and Social Affairs report from 32 enterprises in 2007/8 indicated that the incidence rate was significantly higher in textile factories as compared with other enterprises. According to this report, only Bahir Dar and Kombolcha textile factories constituted of 34.45% of the cases from the total reported injuries [18]. The working conditions of the two factories force workers to expose for different occupational injury risk factors. For example, the working rooms in both factories are too hot; there are high speed rotating and unprotected machines and the working time shift causes workers to have sleep disturbance problem.

These studies done in Ethiopia indicated that there were different causes of occupational injury. According to a study done in eleven urban industries in Addis Ababa, it was indicated that being hit by or against objects and falling were the commonest causes of work-related injuries [12]. Findings of a study done among textile factory workers in Addis Ababa demonstrated that the most frequent causes of occupational injury were machinery 42 (29.4%), and being hit by or against objects 29 (20.3%) [19]. Department of Environmental Health in Ministry of Health in Ethiopia reported that striking (25.5%), falling (12.8%), and flying objects from machines (8.5%) were the major causes of occupational injury [17]. Similarly the Amahara regional BOLSA reported that machinery (36.7%), mishandling (15.3%), falling (14.5%), and hand tools (6.2%) were the commonly complained occupational injury types among manufacturing industrial workers [18].

In most studies, abrasions, cuts, burns, puncture, and fracture were the common injury types among manufacturing industrial workers [11, 18]. The Amahara regional BOLSA 2007/2008 report also indicated that abrasion (62.5%), cuts (12.2%), and punctures (6.7%) were the commonest occupational injury types [6]. The common affected body parts among eleven industrial workers in Addis Ababa were fingers (37.3%) and hands (11.6%) [3]. A study done in Addis Ababa among textile factory workers reported that the most common affected body parts due to work-related injuries were fingers (42%), lower leg (18.95%), and hands (13.3%) [19]. Reports from Department of Environmental Health of Ministry of Health of Ethiopia listed eyes, hands and fingers as the most commonly affected parts of the body [17]. Similarly, a study done among small- and medium-scale factory workers in North Gondar Zone indicated that hands were with the highest frequently affected body parts (30%) followed by fingers (24%) and eyes (19%) [1].

All of the above studies except few were focused on the characterization of occupational injury among industrial workers. However, to solve occupational health and safety problems of the workforce, advanced epidemiological studies are essential for policy makers, public health experts and program implementers. Therefore, this case control study was designed to fill the gap by identifying the determinants of occupational injury among textile factory workers, which, is very important for the development and strengthening of legislations and intervention priorities to safeguard the health and safety of the work force.

## 2. Methods

### 2.1. Study Design and Period

An institutional-based unmatched case control study was done from October 7 to 27, 2009 to identify determinants of occupational injury among textile factory workers.

### 2.2. Study Area

The study was conducted among Bahir Dar and Kombolcha textile factory workers in Amahara regional state. The two institutions are the only textile factories in Amahara regional state which are burdened 34.45% of occupational injuries from the total reported cases in the region in 2007/8 [6]. The two factories were included in the study to get adequate sample size for cases by considering that the two factories were homogeneous for variables under investigation. Bahir Dar textile factory was established in 1966 in Bahir Dar town which is located 565 kilometers away from Addis Ababa with a total of 1246 production site workers (57% males and 43% females). Kombolcha textile factory was established in 1986 in South Wollo Zone, Kombolcha town which is located 380 Kilometers away from Addis Ababa with a total of 1564 production site workers (58% are males and 42% females). In both industries, there is an insurance mechanism for workers that may be injured during work. This encouraged the workers to report every accident during work.

### 2.3. Study Population

All production site workers were included in the study with the following case and control definition. Cases were workers who have experienced occupational injury within one-year period (from September 2008 to September 2009) in textile factories, and the control groups were workers who did not experienced occupational injury within one-year period (from September 2008 to September 2009) in textile factories. To ascertain cases and the control group, both self-reported occupational injury data from the worker and document review from the factories clinic in combination were used to categorize a worker as a case or control group. To be a case, the injury status for cases and noninjury status for controls must be confirmed by both methods to reduce misclassification bias.

### 2.4. Sample Size, Techniques, and Procedures

The sample size was calculated using EPI INFO version 6 statistical software's *statCalc* program for unmatched case control study design. The control group exposure to sleeping disorder (58.4%), lack of training on health and safety (35.3%), and 5 years or less work experience (27.3%) were considered for sample size determination from previous studies [19–21]. From the above determinants, exposure of the control group to sleeping disorder problem (main exposure variable) gave the maximum sample size with assumptions of a one-to-two case-to-control ratio, a minimum detectable odds ratio of 2 and 95% confidence interval, 85% power of the study. Based on the above assumptions, 138 for cases and 276 for the control group with a total of 414 study subjects were calculated. None response rate 10% was added both for cases and controls. Finally a total of 456 study participants (152 cases and 304 controls) were included in the study. Prior to the actual data collection, a baseline survey and factories clinic document review were done for six days to identify source of cases and the control groups. Both document review and self-reported occupational injury status were done to reduce misclassification bias among cases and the control groups. Then a sampling frame was prepared for both cases and the control group based on self-reported data from the worker and document review from the factory clinic results. Then the study subjects were selected by deriving an assumption that determinant factors of occupational injury were homogeneous in the factories.

### 2.5. Data Collection

Data was collected using pretested and structured Amharic version questionnaire via face to face interview of the study participants after getting ethical clearance from responsible bodies and informed verbal consent from study subjects. The questionnaire focused on sociodemographic, behavioral, and environmental variables. Data collection was administered by grade 10 completed students, and two supervisors after two-day training. Job stress, and job satisfaction of workers were assessed using 14 and 12 three-scale item standardized workers response questionnaire, respectively [14]. 

### 2.6. Study Variables

Occupational injury status was the outcome variable and sociodemographic, behavioral, and environmental factors independent variables.

- *Socio demographic factors*: sex, age, religion, ethnicity, marital status, level of education, monthly salary, employment condition, work experience.
- *Work environment determinants*: health and safety information, health and safety training, workplace supervision, working department.
- *Behavioral determinants*: Alcohol consumption, khat chewing, cigarette smoking, sleeping disorder, job satisfy action, job stress, and personal protective equipment use.

### 2.7. Operational Definition

**(i) Occupational Injury**

Any physical injury condition sustained on worker in connection with the performance of his or her work in textile factories [9].

**(ii) Job Satisfaction**

A worker who have scored above or equal to the 90th percentile was considered to have job satisfaction and below the 90th percentile was considered to be dissatisfied by his/her job [14].

**(iii) Job Stress**

A worker who have scored above or equal to 90th percentile was considered to have a problem of job stress and below the 90th percentile was considered to have job stress [14].

**(iv) Health and Safety Information**

A worker who have got any kind of information in-one-year period through any kind of media about health and safety of factory workers.

**(v) Health and Safety Training**

Trainings given to a worker about health and safety to factory workers.

**(vi) Work Place Supervision**

Regular supervisions done by health and safety responsible bodies in the department and working rooms.

**(vii) Working Department**

One of the factor manufacturing units in the department.

**(viii) Khat Chewing**

It is the practice of chewing khat leaves by the worker at least once per week for different purposes.

**(ix) Cigarette Smoking**

In halation of the gases and hydrocarbon vapors generating by slowly burning of cigarettes regularly.

**(x) Personal Protective Equipment (PPE).**

Utilization of the worker-specialized clothing or equipment worn by employees for protection against health and safety hazards at the time of interview. Personal protective equipment is designed to protect many parts of the body, that is, eyes, head, face, hands, feet, and ears.

**(xi) Sleeping Disturbance Problem**

The presence of sleeping problems when the worker is at work in the factory.

### 2.8. Data Management and Quality Control

The questionnaire was prepared originally in English and translated to Amharic. Training of the data collection team with pretesting in 10% of the sample size before the actual survey was made for two days to ensure the possible quality of the data. The principal investigator and supervisors checked and reviewed the filled questionnaires to ensure completeness and consistency of the information collected at each factory.

### 2.9. Data Processing and Analysis

After editing, cleaning, and coding, the data was entered to version 16 SPSS for analysis. Bivariate logistic regression analysis was employed to see association between determinants and occupational injury. Crude odds ratio with confidence intervals and significance level at *P* < 0.05 were used to see the association between determinants and occupational injury. Variables with *P* at <0.2 during the bivariate analysis were included in the multivariate logistic regression analysis to see the interaction effect of confounding variables. Adjusted odds ratio with confidence interval and at *P* < 0.05 as significant level were calculated.

## 3. Results

One hundred fifty two cases and 304 controls were interviewed for this study, and from these, 76.9% of cases and 59.2% controls were male workers. The mean year of work experience for cases was 10.8 and 14.0 for controls and 95.0% of the cases and 93.8% of controls were permanently employed in the factories.

### 3.1. Sociodemographic Determinants

From socio demographic determinant variables, age group at interview, sex and work experience showed statistically significant association with occupational injury in the bivariate analysis. The rest socio demographic variables like religion, ethnicity, marital status, educational level, employment condition, and monthly salary did not show significant association with occupational injury. Only sex and age remained significant in multivariate model, while years with job became nonsignificant (Table 1).

### 3.2. Work Environment Determinants

From work environment determinants, information access to health and safety (COR 1.49, 95% CI 1.01, 2.20), regular work place supervision (COR 1.58, 95% CI 1.07, 2.35), and training on health and safety (COR 2.2, 95% CI 1.45, 3.39) showed significant association with occurrence of occupational injury. But working department did not show a significant association with occupational injury occurrence in the bivariate analysis. After adjusting in the multivariate analysis, training on health and safety remained a significant predictor of occupational injury (AOR 1.85, 95% CI 1.18, 2.91) (Table 2).

### 3.3. Behavioral Determinants

From behavioral determinants, personal protective equipment use (COR 1.77, 95% CI 1.18, 2.64), alcoholic drink consumption (COR 1.68, 95% CI 1.11, 2.55), sleeping disturbance (COR 2.26, 95% CI 1.52, 3.36), job dissatisfaction (COR 1.97, 95% CI 1.09, 4.33), and job stress (COR 2.29, 95% CI 1.23, 4.25) had showed significant association with occupational injury in the bivariate analysis. However, khat chewing (COR 1.27, 95% CI 0.76, 2.12) and cigarette smoking (COR 1.28, 95% CI 0.65, 2.51) did not show significant association with occupational injury. Workers who complained problems of sleeping disturbance were more likely to report two times excess occupational injury compared with workers who did not report problem of sleeping disturbance (AOR 1.99, 95% CI 1.30, 3.04). This study revealed that job stress was the main predictor of occupational injury. Workers who were stressed due to their job were about 2 times more likely to report occupational injury compared with workers who were not stressed due to their job (AOR 2.25, 95% CI 1.15, 4.41) (Table 3).

## 4. Discussion

Studies done in developed and developing countries reported that men had a higher risk of occupational injury than women in manufacturing industries [19, 20]. According to this finding, male workers were about 2.5 times more likely to report occupational injury than female workers (AOR: 2.54, 95% CI 1.58, 4.07). This can be explained due to the following factors high willingness of male workers are more inclined to engage towards risk-taking behavior than female workers [22].

Most study findings at different places by different scholars reported that working at younger age increases the risk of sustaining more occupational injury among factory workers compared with older workers [2, 11, 20]. Similarly this study revealed that workers in age group below 30 years old were about 1.9 times more likely to report occupational injury than workers whose age group was 30 years and above (AOR 1.90, 95% CI 1.22, 2.94). This can be explained as due to the fact that inaccessibility to health and safety information, lack of training on health and safety, less work experience, low level of knowledge and skill towards the work among young workers [1, 11]. But this study contradicts with a case control study done among coal mining industrial workers in India which reported that older age group workers were at higher risk of occupational injury than young age group workers. This result was explained by the investigators as aging would result in a decrease in physical and mental abilities which may in turn alter the quality of work performance and the ability to notice work environment hazards, particularly when the demanding level of the tasks is high [15].

Most occupational health and safety studies conducted in developing countries revealed that increased educational level has been associated with decreased work-related injuries [2, 10, 12, 13]. This is due to the fact that education is more likely to increase workers safety and health practice that can prevent them from occupational injuries [9, 20]. But this study and a cross-sectional study done in Ethiopia among small- and medium-scale factory workers revealed that educational level did not show any statistical significant association with occurrence of occupational injury [11]. This difference may be due to the fact that only education by itself alone cannot reduce occupational injury when the level of hazards is high and the use of reliable techniques and safe work organizations are limited [11].

Literatures indicated that there is a strong relationship between training on health and safety and reduced work accident rates among industrial workers. This is due to the fact that health and safety training could both motivate workers to be safer and instruct them in correct safety behaviors [20]. This study indicated that workers who did not train on health and safety were 1.8 times more likely to report occupational injury than workers who trained last year or before (AOR 1.85, 95% CI 1.18, 2.91). This result was supported by studies done among Assiut spinning factory workers in 2004 in Egypt [21] and among small- and medium-scale factory workers in Ethiopia [11]. These findings were due to the fact that training on health and safety can change both attitude and safety behaviors and on the other side lack of training on health and safety leads to lack of knowhow and job knowledge [2, 21].

Different scholars reported that sleep disturbances such as difficulty in initiating sleep, sleeping poorly at night, sleep insufficiency, and insomnia symptoms are significantly associated with the occurrence of occupational injuries [23]. This study also revealed that workers who complained from sleeping disturbance during work had about two times more likely to report occupational injury than workers who did not report a problem of sleeping disturbance (AOR 1.99, 95% CI 1.30, 3.04). Most occupational health and safety studies conducted in developing and developed countries strongly agreed with this finding [9, 11]. This is due to the fact that workers in textile factories were employed in three shifts with 8 working hour's interval which may disturb the sleeping pattern of workers. These sleeping disturbance problems affect the ability to maintain wakefulness and concentration as well as the ability to assess or watch the work environment and working conditions and perform duties safely.

This study finding indicated that workers who were stressed highly due to their job were more likely to report more than 2.5 times occupational injury compared with their counterparts (AOR 2.25, 95% CI 1.15, 4.41). This result was supported by a case control study done among coal mining industrial workers in India (AOR: 1.83; 95% CI 1.0, 3.4) [15]. Another case control study done among Iranian car manufacturing workers reported that the risk of occupational injury among those with high job stress was significantly higher than those with low job stress (AOR 2.00; 95% CI 1.2, 3.3) [24]. This can explained as job stress can result in physiological and psychological alterations that may increase the likelihood of developing physical and mental problems. These conditions may increase the risk of sustaining more occupational injury among industrial workers [16]. In this we have limitations on measurement of environmental determinant factors like heat, lightening, moisture, and noise level at working site due to lack of measuring instruments.

 From this study, we concluded that being male worker, younger in age, job stress and having sleeping disturbance increases occupational injury and provision of health and safety training can reduce the occurrence of occupational injury. So training of workers on health and safety as well as reducing job stressors and sleep disturbances were recommended.

## Figures and Tables

**Table 1:** Association between sociodemographic variables and occupational injury among textile factory workers in Amhara regional state Ethiopia, October 2009.

Socio-demographic variables	Cases (*n* = 152) No. (%)	Controls (*n* = 304) No. (%)	COR^@^ (95% CI)	AOR^@^ (95% CI)
Sex				
Male	117 (76.97)	180 (59.21)	2.30 (1.48, 3.58)***	2.54 (1.58, 4.07)***
Female	35 (23.03)	124 (40.79)	1.00	1.00
Age group				
<30 years	80 (52.63)	104 (34.21)	2.14 (1.44, 3.18)***	1.90 (1.22, 2.94)**
≥30 years	72 (47.37)	200 (65.79)	1.00	1.00
Religion^@^				
Orthodox	98 (64.47)	176 (57.89)	0.93 (0.22, 3.97)	
Muslim	51 (33.55)	123 (40.46)	0.69 (0.16, 3.00)	
Protestant	3 (1.97)	5 (1.64)	1.00	
Ethnic group^@^				
Amhara	140 (92.11)	282 (92.76)	1.49 (0.15, 14.45)	
Tigre	11 (7.24)	19 (6.25)	1.74 (0.16, 18.80)	
Oromo	1 (0.66)	3 (0.99)	1.00	
Marital status^@^				
Married	99 (65.13)	191 (62.83)	1.04 (0.35, 3.12)	
Single	44 (28.95)	91 (29.93)	0.97 (0.31, 3.00)	
Divorced	4 (2.63)	12 (3.95)	0.67 (0.14, 3.17)	
Widowed	5 (3.29)	10 (3.29)	1.00	
Educational level				
≤grade 8	75 (49.34)	136 (44.74)	1.10 (0.66, 1.86)	1.27 (0.70, 2.33)
Grade 9–12	47 (30.92)	108 (35.53)	0.87 (0.50, 1.52)	0.92 (0.50, 1.71)
Certificate and above	30 (19.74)	60 (19.74)	1.00	1.00
Employment condition				
Employment contract	145 (95.39)	285 (93.75)	1.00	1.00
Temporary contract	7 (4.61)	19 (6.25)	1.38 (0.57, 3.36)	1.45 (0.54, 3.91)
Monthly salary in birr^@^				
≤467 birr per month	80 (52.63)	172 (56.58)	0.85 (0.58, 1.26)	
>467 birr per month	72 (47.37)	132 (43.42)	1.00	
Work experience in years				
5 years and below	60 (39.47)	91 (29.93)	1.53 (1.01, 2.29)*	1.59 (0.86, 2.95)
6 years and above	92 (60.53)	213 (70.07)	1.00	1.00

Note: ^@^not included in multivariate analysis.

Significant at: *P ≤ 0.05, **P ≤ 0.01, ***P ≤ 0.001.

COR^@^: Crude odds ratio and AOR^@^: Adjusted odds ratio.

**Table 2:** Association of occupational injury with environmental determinants among textile factory workers, Amhara regional state, Ethiopia October 2009.

Work environment variables	Case (*n* = 152) No. (%)	Control (*n* = 304) No. (%)	COR^@^ (95% CI)	AOR^@^ (95% CI)
Health and safety information access				
Yes	70 (46.10)	170 (55.92)	1.00	1.00
No	82 (53.97)	134 (44.08)	1.49 (1.01,2.20)*	1.05 (0.68,1.71)
Work place supervision				
Yes	79 (51.97)	192 (63.12)	1.00	1.00
No	73 (48.03)	112 (36.84)	1.58 (1.07,2.35)*	1.12 (0.70,1.78)
Health and safety training				
Yes	41 (26.97)	137 (45.07)	1.00	1.00
No	111 (73.03)	167 (54.93)	2.22 (1.45,3.39)***	1.85 (1.18,2.91)**
Working department^@^				
Spinning	64 (42.11)	137 (45.07)	1.00	
Weaving	48 (31.58)	90 (29.61)	1.14 (0.72,1.18)	
Finishing	26 (17.11)	54 (17.76)	1.03 (0.59,1.79)	
Engineering	14 (9.21)	23 (7.57)	1.30 (0.69,1.79)	

Note: Significant at, *P ≤ 0.05, **P ≤ 0.01, ***P ≤ 0.001.

^@^Not included for multivariate analysis.

COR^@^: Crude odds ratio and AOR^@^: Adjusted odds ratio.

**Table 3:** Association of occupational injury with behavioral determinants among textile factory workers, Amhara regional state, Ethiopia October 2009.

Behavioral variables	Case (*n* = 152) No. (%)	Control (*n* = 304) No. (%)	COR^@^ (95% CI)	AOR^@^ (95% CI)
PPE use				
Yes	54 (35.53)	150 (49.34)	1.00	1.00
No	98 (64.47)	154 (50.67)	1.77 (1.18, 2.64)**	1.31 (0.82, 2.10)
Alcohol use				
Yes	57 (35.5)	80 (26.32)	1.68 (1.108, 2.55)*	1.40 (0.89, 2.21)
No	95 (62.5)	224 (73.68)	1.00	1.00
Khat chewing^@^				
Yes	28 (18.42)	46 (15.13)	1.27 (0.76, 2.12)	
No	124 (81.59)	258 (84.87)	1.00	
Cigarette smoking^@^				
Yes	15 (9.87)	24 (7.89)	1.28 (0.65, 2.51)	
No	137 (90.13)	280 (92.11)	1.00	
Sleeping disturbance				
Yes	93 (61.18)	125 (41.12)	2.26 (1.52, 3.36)***	1.99 (1.03, 3.04)**
No	59 (38.82)	179 (58.88)	1.00	1.00
Job stress				
Yes	23 (15.13)	22 (7.24)	2.28 (1.23, 4.25)***	2.25 (1.15, 4.14)**
No	129 (84.87)	282 (92.76)	1.00	1.00
Job satisfaction				
Yes	15 (9.87)	54 (17.76)	1.00	1.00
No	137 (90.13)	250 (82.24)	1.97 (1.09, 4.33)*	1.49 (0.76, 2.93)

Note: Significant at, *P ≤ 0.05, **P ≤ 0.01, ***P ≤ 0.001.

^@^Not included for multivariate analysis.

COR^@^: Crude odds ratio and AOR^@^: Adjusted odds ratio.
